# Molecular convergent and parallel evolution among four high-elevation anuran species from the Tibetan region

**DOI:** 10.1186/s12864-020-07269-4

**Published:** 2020-11-27

**Authors:** Bin Lu, Hong Jin, Jinzhong Fu

**Affiliations:** 1 Chengdu Institute of Biology, Chinese Academy of Sciences, Chengdu, China; 2 University of the Chinese Academy of Sciences, Beijing, China; 3 Department of Integrative Biology, University of Guelph, Guelph, Canada

**Keywords:** Molecular convergent evolution, High-elevation adaptation, Amphibian, Tibet, Positive selection, HSP90AA1

## Abstract

**Background:**

To date, evidence for the relative prevalence or rarity of molecular convergent and parallel evolution is conflicting, and understanding of how these processes contribute to adaptation is limited. We compared four high-elevation anuran species (*Bufo tibetanus*, *Nanorana parkeri*, *Rana kukunoris* and *Scutiger boulengeri*) from the Tibetan region, and examined convergent and parallel amino acid substitutions between them and how they may have contributed to high-elevation adaptation.

**Results:**

Genomic data of the four high-elevation species and eight of their low-elevation close relatives were gathered. A total of 1098 orthologs shared by all species were identified. We first conducted pairwise comparisons using Zhang and Kumar’s test. Then, the *R*_*conv*_ index was calculated and convergence/divergence correlation plotting was conducted. Furthermore, genes under positive selection and with elevated evolutionary rate were examined. We detected a large number of amino acid sites with convergent or parallel substitutions. Several pairs of high-elevation species, in particular, *R. kukunoris* vs *N. parkeri* and *B. tibetanus* vs *S. boulengeri*, had excessive amounts of convergent substitutions compared to neutral expectation. Nevertheless, these sites were mostly concentrated in a small number of genes (3–32), and no genome-wide convergence was detected. Furthermore, the majority of these convergent genes were neither under detectable positive selection nor had elevated evolutionary rates, although functional prediction analysis suggested some of the convergent genes could potentially contribute to high-elevation adaptation.

**Conclusions:**

There is a substantial amount of convergent evolution at the amino-acid level among high-elevation amphibians, although these sites are concentrated in a few genes, not widespread across the genomes. This may attribute to the fact that all the target species are from the same environment. The relative prevalence of convergent substitutions among high-elevation amphibians provides an excellent opportunity for further study of molecular convergent evolution.

**Supplementary Information:**

The online version contains supplementary material available at 10.1186/s12864-020-07269-4.

## Background

Convergent and parallel evolution at a molecular level (collectively referred as molecular convergent evolution) have attracted tremendous interest in the last few years. Although biologists have long considered phenotypic convergence the best evidence for adaptive evolution, our understanding of the underlying genetic mechanisms has just begun [1–4]. These processes have significant implications in understanding the repeatability and predictability of evolution, and are of fundamental importance in biology ([1, 5]; also see review [2, 6]). Furthermore, given there are usually many potential genetic solutions to a problem, what causes molecular convergent evolution and how frequently it occurs in natural populations remain major topics in evolutionary biology [6]. Two levels of molecular convergent evolution are often examined. At the gene level, different organisms may modify the same gene to solve the same problem even though the modifications (mutations) themselves are not necessarily the same [7]. At the amino acid level, different organisms may share the same amino acid substitutions [8]. We define amino acid level convergent substitutions as independent changes from different ancestral amino acids to the same derived amino acid, when orthologous proteins between two or more species are compared. Similarly, parallel substitutions refer to independent changes derived from the same ancestral amino acid [6].

One major controversial issue regarding molecular convergent evolution at the amino acid level is whether it is commonplace across the genome or restricted to a few particular loci. Most classic studies focused on a few specialized genes and implied that it was restricted. The hearing genes (e.g. *prestin*) between echolocating bats and dolphins are probably the best-known cases [9–11]. Other recent studies also suggest that molecular convergent evolution is restricted to a few loci (e.g. Opsins in insects [12], ribonuclease genes in monkeys [13], RH1 in bats [14], auditory genes in bats and whales [15, 16], TTX resistance in garter snakes and newts [17, 18]). The general understanding is that molecular convergent evolution is rare in natural populations, given evolution more likely uses different genetic mechanisms in different lineages ([9, 19], also see review [2, 6]). Furthermore, experimental evolution on *E. coli* also demonstrated that convergent evolution at the amino acid level is rare, although it may not be rare at the gene level [1]. On the other hand, Rokkas and Carroll [20] argued that there was a high frequency of parallel amino-acid substitutions in eukaryotic proteins. More recently, Parker et al. [21] detected convergent amino acid substitutions that were widespread and continuously distributed in genomes of echolocating mammals (but see [22, 23]). Foote et al. [24] compared three groups of marine mammals and concluded that molecular convergent substitutions were relatively common, although substitutions that could be linked to phenotypic convergence were rare. Similarly, Hu et al. [25] found a large number of molecular convergent genes between the giant panda and red panda. Evidently, the re-use of genes in natural populations is common, but the frequency of re-use of the same point mutation remains disputable.

Whether molecular convergent evolution results from neutral evolution or is associated with adaptive evolution is another controversial issue. Phenotypic convergence has long been associated with adaptation [26, 27]. Many works have explicitly associated molecular convergent evolution with adaptive evolution [1, 19]. Theoretical modeling work also demonstrated that the probability of molecular convergent evolution indeed increases under positive selection [28]. Two recent studies examined molecular convergent evolution only among genes under positive selection, implying an association [24, 25]. On the other hand, several other studies, both empirical and model based, suggested that neutral evolution could sufficiently explain most observed molecular convergent events [22, 23, 29].

Organisms living at high-elevation environments provide excellent model systems for studying molecular convergent evolution and its association with adaptation. At high-elevations, organisms are exposed to severe physiological stressors, particularly those related to hypoxia, low ambient temperature, high UV radiation, and high seasonal variability [30, 31]. Distantly-related organisms experiencing the same selection process have a high probability of independently evolving similar phenotypic adaptations. Some of them are likely based on the same genetic architecture, similar to the convergent evolution between marine mammals [24]. This ecological context is essential in understanding convergent adaptive evolution. Furthermore, simultaneous adaptive responses to multiple stressors at high-elevations likely involve interactions and trade-offs between genes and pathways [31]. As such, deleterious pleiotropy and epistasis, which are often considered the leading causes of molecular convergent evolution [6], are expected to be strong; therefore, molecular convergent evolution is expected to be common. Indeed, several previous studies have identified molecular convergent evolution among high-elevation dwellers. Sun et al. [32] reported genetic convergence between high-elevation frogs and lizards at a functional level, and others reported protein coding genes with convergent and parallel amino-acid sites between various high-elevation vertebrates (e.g. HIF genes, Hb genes, MYBPC2, HSP90AA1 [33–36];). Several anuran species thrive on the Tibetan Plateau (elevation > 3000 m); this is extraordinary considering that amphibians are ectothermic and are most successful in the tropical zone. Recent studies have revealed many positively selected genes, which have likely participated in the adaptation process [37, 38].

In this study, we compared the genomic data of four anuran species living at high elevations around the Tibetan Plateau region. These species belong to different families and have diverged between 60 and 165 Mya [39]. Specifically, we would like to address these questions: 1) Is molecular convergent evolution widespread in the genome or restricted to a few particular loci? 2) What types of genes are involved in molecular convergent evolution? 3) Are these convergent genes associated with adaptation to high-elevation environments? We gathered both genome and transcriptome data for 13 species, including the four high-elevation anuran species and eight of their low-elevation close relatives and one outgroup species. Both convergent and parallel amino acid substitutions were tested between the high-elevation species. Additionally, signals of positive selection among these high-elevation species were also examined. We further compared the results from the two sets of analyses and inferred potential roles of these related genes in the process of high-elevation adaptation.

## Results

### Species selection and data

**Fig. 1:**
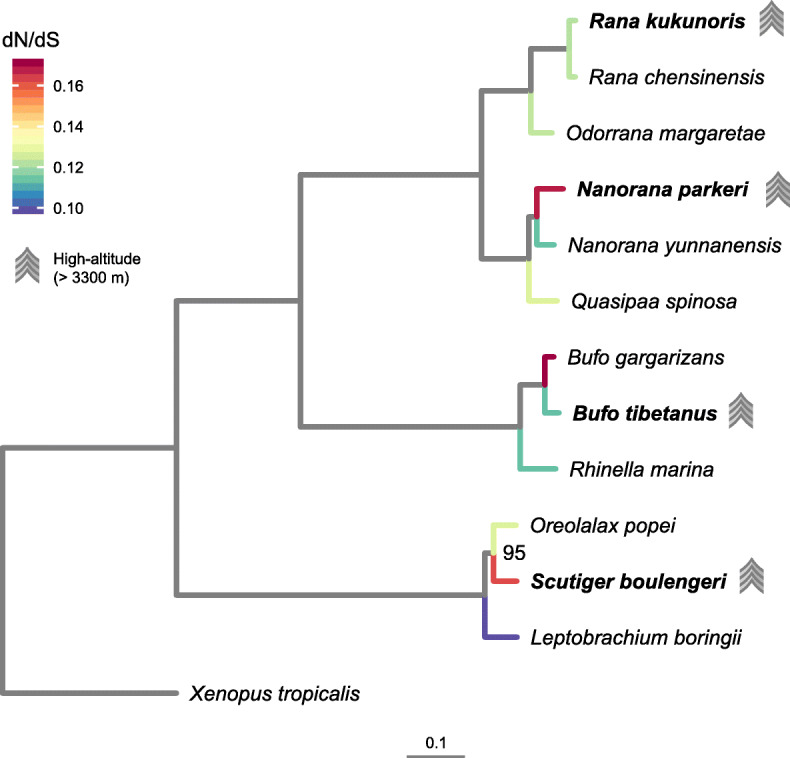
Phylogeny of the 13 species examined in this study. The tree is derived from a maximum likelihood analysis of fourfold degenerate (4D) sites of a concatenated dataset of 1198 orthologous genes (dataset 1). Bootstrap proportions are 100 for all nodes except the sister-group relationship between *S. boulengeri* and *O. popei*, which is 95. High-elevation species are marked with symbols. Branch colors represent dN/dS values for external nodes

A total of 13 species were included in our analysis (Fig. 1). Four high-elevation anuran species, *Bufo tibetanus*, *Nanorana parkeri*, *Rana kukunoris* and *Scutiger boulengeri* were first selected. These species are primarily distributed at elevations higher than 3000 m while *N. parkeri* and *S. boulengeri* reach above 5000 m [40]. Based on established phylogenies [41–44], the sister-group species or a closely related low-elevation relative of each high-elevation species (*B. gargarizans, N. yunnanensis, R. chensinensis,* and *Oreolalax popei*) was selected. A second low-elevation close relative to each high elevation species (*Rhinella marina, Quasipaa spinosa, Odorrana margaretae,* and *Leptobrachium boringii*) was also included in our analysis to help with estimating ancestral sequences. Thus, a total of four sets of species were selected and we used *Xenopus tropicalis* as an outgroup (Fig. 1).

Genome data for three species and transcriptome data for six species were obtained from published sources, and we sequenced the transcriptomes for four species (*B. tibetanus, O. popei, Q. spinosa,* and *S. boulengeri*) in this study. Summary information of the transcriptome assemblies used in this study and related experimental data are presented in Additional file Table S1.

Putative orthologous genes were identified using the best reciprocal blast hits (BRBH) method. We constructed seven datasets for various analyses. Dataset 1 included all 13 species and 1098 orthologs that were shared by all of them. All alignments in this dataset passed through several quality-control filtering processes and we primarily used this dataset for our analysis. Datasets 2–7 each included two sets of species and 3158 to 4182 orthologs. They were designed for pairwise comparison only, which maximized the number of orthologs in each comparison. Table 1 and Additional file Table S2 present summary information for datasets 1–7.

**Table 1:** Summarized results from Zhang and Kumar’s (1997) test for dataset 1 (1098 orthologs). For each pairwise comparison, only the high-elevation species are listed. Refer to Fig. 1 for a complete list of species and their relationships

Species set	Number of convergent genes	Number of parallel genes	Total number of convergent/parallel genes
*N. parkeri* vs *B. tibetanus*	4	10	12
*N. parkeri* vs *S. boulengeri*	7	26	32
*R.kukunoris* vs *B. tibetanus*	0	3	3
*R. kukunoris* vs *N. parkeri*	3	5	6
*R. kukunoris* vs *S. boulengeri*	1	6	6
*B. tibetanus* vs *S. boulengeri*	1	13	13

### Species phylogeny

We first constructed a phylogenetic tree for the 13 species, which was used for all downstream analysis. To reduce effects of several confounding factors in phylogenomic reconstruction, only fourfold degenerate (4D) sites from the dataset 1 were used [45, 46]. A total of 60,509 base pairs were included after removing gaps. The best-fit partitioning schemes divided the supermatrix into seven subsets. The GTR + G model was selected for each subset. The maximum likelihood (ML) analysis resulted in one optimal tree (Fig. 1). All nodes received 100% bootstrap support, except the sister-group relationship between *S. boulengeri* and *O. popei* (bootstrap proportion = 0.95). All relationships were consistent with established anuran phylogenies [47, 48].

### Genes with molecular convergent evolution

We first conducted pairwise comparisons between any two of the four high-elevation anurans using Zhang and Kumar’s test [49]. Table 1 presents the summarized results from all six pairwise comparisons based on dataset 1 (1098 orthologs), and a complete list of convergent and parallel sites and other detailed information is provided in Additional file Table S3. A total of 57 unique genes were identified as having one or more convergent or parallel amino-acid substitutions between two species (*p* < 0.05 for both Poisson and JTT models). In five of the six pairwise comparisons, the detected convergent and parallel sites were concentrated among several genes (13 genes or fewer; Table 1); in the case of *N. parkeri* vs *S. boulengeri*, they were more widespread across a large number of genes (32 genes; Table 1). The summarized results from additional pairwise comparisons on datasets 2–7 are presented in Additional file Table S2. These comparisons included many more orthologs (3158 to 4182 orthologs) than those based on dataset 1. Similarly, 153 convergent and parallel genes were identified, and most convergent and parallel sites were concentrated among a small number of genes.

**Fig. 2:**
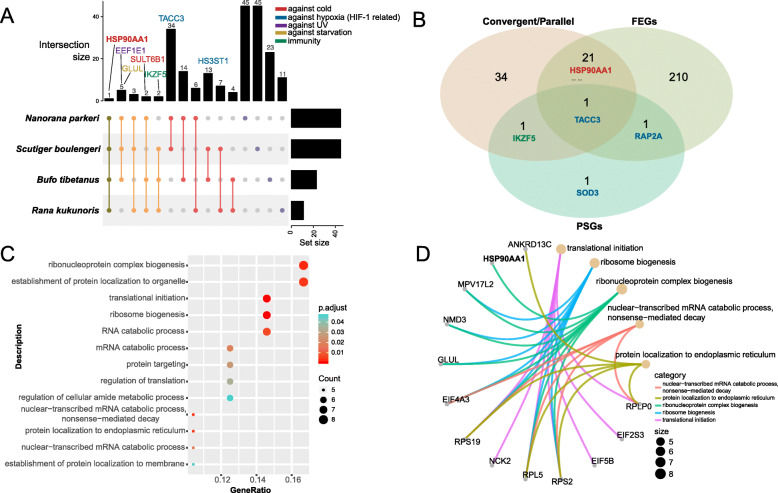
The distribution of genes involving molecular convergent evolution, positive selection, and accelerated evolution. **a** An UpSet diagram to visualize intersections of convergent genes among four high-elevation anuran species. Several convergent genes with functions of immunity, against cold, hypoxia, UV and starvation were marked in different colors. **b** The Venn diagram of positively selected genes (PSGs) identified by CODEML, fast-evolving genes (FEGs), and convergent genes. TACC3 is a PSG, a FEG, and a convergent gene. **c** Enriched biological process (BP) terms of the convergent genes. Only GOs with convergent gene ratio greater than 0.1 were presented. **d** The network of enriched BP terms and related genes

Several genes were detected as having convergent or parallel sites in more than one set of pairwise comparison (Additional file Table S3). Eight convergent genes were shared by three high-elevation species (ANXA5, EEF1E1, GLUL, IKZF5, RDH16, STARD10, SULT6B1, and TMEM238), and one gene (HSP90AA1) was shared by all four high-altitude species. An UpSet diagram presents these overlap relationships (Fig. 2a). The HSP90AA1 gene is of particular interest. We detected 16 parallel sites between *N. parkeri* and *R. kukunoris*, and one parallel substitution in four of the other five pairwise comparisons: *R. kukunoris* vs. *B. tibetanus*, *N. parkeri* vs. *S. boulengeri*, *R. kukunoris* vs. *S. boulengeri*, and *B. tibetanus* vs. *S. boulengeri*. A more detailed examination revealed that no amino acid site was shared by all four high-altitude species, but one site (V186I) was shared by three species (*N. parkeri, R. kukunoris,* and *S. boulengeri*). Furthermore, we tested this gene for potential convergent and parallel evolution between the low-elevation species. Despite the large number of parallel sites (=16) between *N. parkeri* and *R. kukunoris*, we detected no convergent or parallel sites between their low-elevation sister-group species (*N. yunnanensis* and *R. chensinensis*). We also searched for convergent and parallel sites between *N. parkeri* and the three low-elevation sister-group species of other species sets, as well as between *R. kukunoris* and the three low-elevation sister-group species of other species sets. No significant amount of convergent and parallel sites were detected except when paired with *B. gargarizans*. Noticeably, some populations of *B. gargarizans* occur in high-elevation areas [40].

Several genes possessed particularly large numbers of convergent and parallel sites (Additional file Table S3). SULT6B1, a sulfotransferase involved in the metabolism of thyroxine [50], had 56 parallel and 3 convergent substitutions between *R. kukunoris* and *N. parkeri*, and one parallel substitution between *N. parkeri* and *B. tibetanus*. Another sulfotransferase, HS3ST1, had 52 parallel and 11 convergent sites between *B. tibetanus* and *S. boulengeri*. GLUL, a glutamine synthetase involved in the response to glucose and starvation, had 21 parallel and 7 convergent changes between *N. parkeri* and *B. tibetanus*, and 1 parallel site between *B. tibetanus* and *S. boulengeri*.

**Fig. 3:**
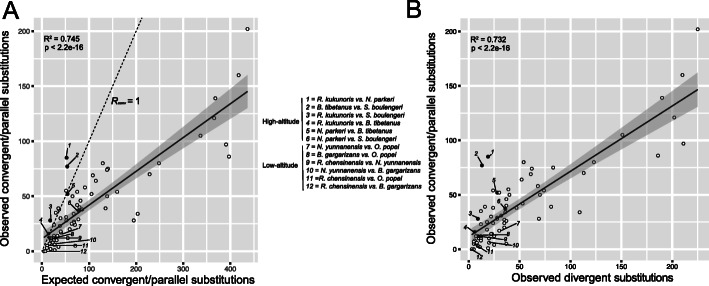
**a** Positive correlation between observed convergent/parallel substitutions and expected convergent/parallel substitutions. **b** Positive correlation between observed convergent/parallel substitutions and observed divergent substitutions. A total of 74 pairwise comparisons are made (=74 data points). Lines present the best-fit lines and grey bands represent 95% confidence interval. Dashed line in (**a**) represents *R*_*conv*_ = 1 (observed convergent/parallel substitutions = expected convergent/parallel substitutions). All six high-altitude pairs are in solid circles

We also examined whether convergent and parallel amino acid substitutions among these high-elevation anurans were more prevalent than neutral expectations using the *R*_*conv*_ index described by Zou and Zhang [29]. *R*_*conv*_ is the ratio between the observed numbers of convergent and parallel sites and the expected numbers under a neutral model of amino acid substitution. We calculated *R*_*conv*_ for all species pairs in dataset 1 using the JTT-f_site_ model. For high-elevation anurans, the ratio was greater than 1.0 in four of the six pairs and greater than 1.5 in two pairs (Table 2; Fig. 3a). In contrast, the *R*_*conv*_ values between the low-elevation species were mostly below 0.75. The *R*_*conv*_ for high-elevation anurans was significantly greater than that of low-elevation species (Wilcox test, *p* = 0.0065). Furthermore, the observed numbers of convergent and parallel sites were significantly correlated with the expected numbers of convergent and parallel sites (Fig. 3a; *R*^*2*^ = 0.745, *p* = 2.2e-16). Nevertheless, four pairs of the low elevation species pairs also showed excessive convergent and parallel sites compared to random expectation (Fig. 3a), including *R. kukunoris* vs *Q. spinosa* (obs: exp. = 55: 51.2), *Q. spinosa* vs *B. tibetanus* (obs: exp. = 38: 36.6), *R. chensinensis* vs *R. marina* (obs: exp. = 35: 29.4), and *R. chensinensis* vs *Q. spinosa* (obs: exp. = 39: 35.0). This could be caused by convergent evolution on other aspects of biology of the related species, and may not be related to high-elevation adaptation.

**Table 2:** Observed numbers of convergent and parallel sites and expected numbers of convergent and parallel sites under neutral evolution. JTT-f_site_ model of amino acid substitution is used and all numbers are calculated using dataset 1. For low-elevation species, only sister-group species of the high-elevation species (Fig. 1) are presented here

	Species pair	Observed convergent and parallel sites	Expected convergent and parallel sites	*R*_*conv*_
High	*B. tibetanus-S. boulengeri*	77	53.794	1.431
	*N. parkeri-B. tibetanus*	52	54.297	0.958
	*N. parkeri-S. boulengeri*	37	77.863	0.475
	*R. kukunoris-B. tibetanus*	16	14.012	1.142
	*R. kukunoris-N. parkeri*	85	52.357	1.623
	*R. kukunoris-S. boulengeri*	28	17.619	1.589
Low	*B. gargarizans-O. popei*	12	24.961	0.481
	*N. yunnanensis-O. popei*	17	40.400	0.421
	*N. yunnanensis-B. gargarizans*	6	26.990	0.222
	*R. chensinensis-O. popei*	5	11.201	0.446
	*R. chensinensis-B. gargarizans*	0	7.214	0.000
	*R. chensinensis-N. yunnanensis*	12	17.004	0.706

The observed numbers of molecular convergent sites are expected to be positively correlated with the numbers of divergent sites [19, 22]. Indeed, we detected a significantly positive correlation between them using dataset 1 (Fig. 3b; *R*^*2*^ = 0.732, *p* = 2.2e-16). The best-fit line may serve as the null expectation under neutral evolution. For all six pairs of high-elevation species, the data points were above the line, although two of them were within the confidence interval (Fig. 3b). Two high-elevation species pairs had particularly high convergence to divergence ratio: 85 convergent and parallel substitutions between *R. kukunoris* and *N. parkeri* with 19 divergent substitutions, 77 convergent and parallel substitutions between *B. tibetanus* and *S. boulengeri* with 13 divergent substitutions (data point 1 and 2; Fig. 3b). Overall, the observed convergent and parallel number in high-elevation anuran species was significantly greater than that of low-elevation counterparts (Wilcox test, *p* = 0.004).

### Genes under positive selection and fast evolving genes

Molecular convergent evolution is often associated with positive selection and adaptation [1, 19], and therefore, we tested genes with positive selection (PSGs) from dataset 1. Furthermore, we examined genes with accelerated evolutionary rates (fast-evolving genes; FEGs). Fast-evolving genes are often associated with positive selection, although relaxed negative selection can also elevate evolutionary rates [51, 52].

The dN/dS ratio and the branch-site model A implemented in CODEML (in PAML, [53]) were used for detecting PSGs. We initially identified 27 genes as potential PSGs (relaxed) within the high-elevation anurans (Additional file Table S4, *p* < 0.05). After adjustment for multiple tests using FDR, only four genes were identified as having significant signal of positive selection (PSGs strict), including RAP2A, TACC3, IKZF5, and SOD3.

For identifying FEGs, we first estimated absolute evolutionary rates for all species using 4D sites, 2nd codon position sites, and lineage-specific dN/dS ratio, which served as background. Table 3 presents rate estimates for each species. At the species level, there was no clear trend, and high-elevation species did not always have higher evolutionary rates. For example, in species sets 2 and 4, high-elevation species had much higher dN/dS ratios than low-elevation ones, but in the other species sets, high-elevation species had lower dN/dS ratios than their low-elevation relatives (Table 3). We further investigated the relationship between evolutionary rate and upper elevation limit. No significant correlation was detected for the 4D site rate (Pearson’s correlation coefficients r = 0.207, *p* = 0.518) and the 2nd codon site rate (r = 0.321, *p* = 0.310; Additional file Figure S1). The dN/dS ratio had a marginally significant correlation with upper elevation limit (r = 0.550, *p* = 0.064; Additional file Figure S1). Finally, we used the one-ratio branch model and multi-ratio branch model in CODEML and a likelihood ratio test to identify fast-evolving genes [54]. A total of 294 genes (relaxed) were identified as potential FEGs in high-elevation species; and 233 genes (strict) remained on the list after FDR adjustment (Additional file Table S5). Two PSGs (strict), RAP2A and TACC3, were also FEGs (strict). Interestingly, several sets of genes from the same family displayed similar fast-evolving character, e.g. heat shock proteins (HSP90AA1, HSPD1, HSPA5 and HSPA9) and proton-transporting ATPase (ATP6V1G1 and ATP6V1H).

**Table 3:** The estimated absolute rates of evolution, dn/ds ratio, and altitudinal range of all 13 anuran species in this study. The high-elevation species are in bold. For the absolute rate of fourfold degenerate (4D) sites and second codon position sites, the unit is number of substitutions per site per million years. The altitudinal range data were collected from Fei et al. [40], Amphibian Species of the World, AmphibiaWeb, and IUCN Red list

Species set	4D site rate	2nd codon rate	dN/dS ratio	Elevational range (m)
**1** ***Bufo tibetanus***	0.003692	0.00039	0.1131	2300–4300
*Bufo gargarizans*	0.002331	0.000491	0.1712	0–2700
*Rhinella marina*	0.002223	0.000176	0.1134	0–1600
**2** ***Nanorana parkeri***	0.00194	0.000267	0.168	2850–5000
*Nanorana yunnanensis*	0.001272	0.000112	0.1135	1500–2400
*Quasipaa spinosa*	0.001591	0.000162	0.1269	200–1500
**3** ***Rana kukunoris***	0.00268	0.000182	0.122	2800–4000
*Rana chensinensis*	0.002536	0.0002	0.123	600–1300
*Odorrana margaretae*	0.001295	0.000112	0.1235	390–1500
**4** ***Scutiger boulengeri***	0.000854	0.000135	0.162	3300–5270
*Oreolalax popei*	0.000825	0.000101	0.1269	900–2000
*Leptobrachium boringii*	0.001112	0.000102	0.0989	700–1700

Not surprisingly, many convergent genes were also PSGs and FEGs. The overlapping relationship among convergent genes, FEGs, and PSGs are presented in Fig. 2b. A total of 22 genes were both FEGs and convergent genes. Three of the four strict PSGs (IKZF5, SOD3 and TACC3) were also convergent genes. Most noticeably, the TACC3 gene, a co-activator for both HIF-1 and HIF-2 complexes, was a PSG, a FEG, and a convergent gene.

We further performed a site-specific positive selection test and assessed how many of the convergent and parallel amino acid substitutions were a product of positive selection. We used the mixed effects model of evolution method (MEME in HyPhy, [55]) and identified 29 sites that were under positive selection from the 57 convergent genes (*p* < 0.05; Additional file Table S6). These sites were distributed in 23 genes, including several genes that are potentially linked to high-elevation adaptation (e.g. HSP90AA1, TACC3, and SULT6B1). Nevertheless, only three of these positively selected sites were convergent or parallel sites.

### Functional prediction

An enrichment analysis of the 57 convergent genes from the pairwise comparison was performed. Functional categories including ribonucleoprotein complex biogenesis and establishment of protein localization to organelle in Biological Processes (BP), ubiquitin protein ligase binding and enzyme inhibitor activity in Molecular Function (MF) ranked high on the list according to gene ratio (Fig. 2c; Additional file Table S7). A similar enrichment analysis of the 153 convergent genes from datasets 2–7 yielded similar results (Additional file Table S8). Furthermore, several convergent genes have multiple functions that were involved in multiple enriched functional categories. For example, GLUL is responsible for ribosome biogenesis and also for ribonucleoprotein complex biogenesis. A network of several enriched BP terms and related genes is presented in Fig. 2d.

An enrichment analysis was also performed for the 233 FEGs (strict). Several functional terms in biological processes, such as response to hypoxia and UV, and in molecular function, such as ATPase activity, were significantly enriched with multiple FEGs (Additional file Table S9).

## Discussion

The four high-elevation species included in this study belong to four distantly related amphibian families (diverged 60–165 Mya, [39]); therefore, standing genetic variation can be ruled out as a probable cause of molecular convergence [2, 56]. Additionally, although amphibians are known to hybridize frequently, crosses between families in natural populations have never been reported, and therefore, horizontal gene introgression among these high-elevation species is unlikely. Furthermore, all four high-elevation species are deeply nested within their low-elevation relatives (Fig. 1 [41–44];), and they likely all evolved from low-elevation ancestors. Therefore, the observed identical de novo amino acid substitutions have likely occurred independently in each lineage during evolution from their respective low-elevation ancestors.

No widespread molecular convergent evolution across the genomes was detected. One of the pairwise comparisons (*N. parkeri* and *S. boulengeri*) using Zhang and Kumar’s test detected a large number of convergent and parallel sites across a relatively large number of genes (32 genes; Table 1), which suggests widespread molecular convergent evolution. However, further tests (Zou and Zhang’s R_conv_, convergence/divergence plotting; Table 2, Fig. 3) revealed no significant increase in convergent evolution compared to neutral expectation between this pair of species. The JTT-f_gene_ model used in Zhang and Kumar’s test is known to be sensitive [29]. This widespread molecular convergent evolution is likely the result of a false positive. For all other pairwise comparisons (e.g. *B. tibetanus* vs *S. boulengeri*) with a large number of convergent and parallel sites, these sites are concentrated within a few genes (3–13 genes, Table 1).

Nevertheless, we detected a substantial amount of molecular convergent evolution among high-elevation amphibians (*R*_*conv*_ = 1.623, Table 2; Fig. 3). This is different from what several recent studies have suggested [22, 29]. The echolocating bat-dolphin system is arguably the best system for studying convergent evolution. Upon re-analyzing data from Parker et al. [21], Thomas and Hahn [22] plotted the number of convergent substitutions against the number of divergent substitutions among nine mammal species. With a strong positive correlation (*R*^*2*^ = 0.8807), the observed number of convergent substitutions between echolocating bats and dolphins is almost exactly as expected under random chance (Fig. 1 in [22]). In our case, the positive correlation is similarly strong (*R*^*2*^ = 0.732), but all high-species elevation pairs are above the best-fit line, and two pairs (*R. kukunoris* vs *N. parkeri* and *B. tibetanus* vs *S. boulengeri*) have substantially more convergent substitutions than random expectation (Fig. 3b, data points 1 & 2). One possible cause of the discrepancy between our study and early studies is the ecological context of our study. We compared species from the same environment (high elevations); these species have been exposed to the same environmental challenges and have likely experienced similar selection processes. Furthermore, high-elevation adaptation likely involves interactions and trade-offs between multiple genes [31], and deleterious pleiotropy and epistasis, which cause convergent evolution, are likely enhanced. Echolocating bats and dolphins have vastly different environments, despite sharing one interesting characteristic. Similarly, the comparison between *Drosophila* species and mammals in Zou and Zhang [29] does not have a specific ecological context. The lack of ecological context between study species may have produced the relatively low observed number of convergent and parallel substitutions (*Drosophila R*_*conv*_ = 0.31–0.61; mammals *R*_*conv*_ = 0.1–1.2), and hence a lack of excessive molecular convergent evolution. Additionally, two recent genome-wide examinations of molecular convergent evolution examined only genes under positive selection [24, 25], probably under the assumption that convergent evolution is more likely to occur within these genes. Although the assumption may be biologically sound, the practice effectively reduces the scope of their examination. In our case, the majority of convergent genes do not have clear indications of being under positive selection (Fig. 2b; Additional file Table S6).

The repeated use of genes is clearly more frequent than the repeated use of amino acids. We observed a large number of genes involved in molecular convergent evolution, and nine genes are involved in convergent evolution among three or four species (Fig. 2a). Nevertheless, we observed only one case of re-using the same amino acid among three species. At one site of the HSP90AA1 gene (V186I), three high-elevation species share an exact de novo amino acid substitution. Zhu et al. [57] reported a similarly rare case; they experimentally confirmed two identical parallel substitutions that contributed to convergent increases in Hb–O2 affinity in co-distributed high-altitude birds. In this regard, our conclusion is in line with early studies [7, 24, 58, 59].

The vast majority of convergent sites and genes do not bear signals of positive selection. Convergent evolution has long been considered the best evidence for adaptive evolution [1, 19, 26, 27], and yet, we failed to detect a clear association between them. There are several potential causes. First, it is expected that a large number of convergent sites are the result of random chance [22, 23]. Empirical data also demonstrated that the majority of convergent amino acid substitutions are functionally inconsequential [8]. Second, most of the current positive selection detection methods are conservative and we may be missing much adaptive evolution at the molecular level [60]. For example, CODEML requires a chronic accumulation of non-synonymous substitutions and usually does not detect an overall pattern of positive selection if only 1–2 sites are under positive selection. On the other hand, tests for molecular convergent evolution could be too liberal [22, 23, 29]. This is a controversial and important issue and more research is certainly needed. We, however, detected three convergent sites that have experienced positive selection (Additional file Table S6). What functional consequences these amino-acid substitutions may have, if any, remains to be examined.

Many detected convergent genes have functions that are essential to high-elevation adaptation, suggesting they could potentially be involved in high-elevation adaptation (but see [61]). Genes associated with energy metabolism and nutrition have been suggested to be involved in the adaptive process, particularly in poikilothermic animals [37, 38]. HSP90AA1 is one of these genes and deserves more attention. Molecular convergent evolution was previously reported between two high-elevation amphibian species *R. kukunoris* and *N. parkeri* [36]. Similar to the previous study, we found a large number of convergent and parallel sites among high-elevation species. It also has an elevated evolutionary rate. The HSP90 gene family plays a number of important roles, including response to cold and heat stress, most of the ATPase activity, assisting in folding, maintenance, and degradation of proteins, as well as facilitating cell signaling. This gene is currently under intensive study. Several other convergent genes, such as SULT6B1 and GLUL, are also involved in energy metabolism [50, 62]. Response to hypoxia is another key function in high-elevation adaptation [30, 31]. TACC3 is a convergent gene that has an elevated evolutionary rate and is also under positive selection. It evidently regulates transactivation of hypoxia-inducible factor (HIF), which is a key transcriptional effector of the hypoxia response and promotes acclamation to low oxygen levels [63, 64]. Several other genes, such as RAP2A (FEG and PSG) and HS3ST1, are also part of response to hypoxia [65–67]. Enhanced UV tolerance and DNA repair capacity may also represent adaptation at high elevations. SOD3 (PSG) is an antioxidant enzyme that catalyzes the degradation of superoxide radicals into hydrogen peroxide and oxygen, which may protect the brain, lungs, and other tissues from oxidative stress. SOD3 is also involved in the biological process of response to sustained hypoxia, and can protect UV-induced injury of the skin [68]. The IKZF5 gene encodes DNA-binding protein and is a transcriptional repressor. It bears clear evidence for both convergent evolution and positive selection (Fig. 2a, b), suggesting that it may play an important role in high-elevation adaptation, and yet, we known little of its functions in any physiological processes of amphibians. This fact highlights the gross deficiency of our understanding of amphibian physiology.

There are several caveats in our study. Firstly, we used fairly disparate datasets and there are quality differences in sequencing, assembly, and gene annotation between them. To minimize the impacts of these differences, we used genome sequence data whenever they were available. For all transcriptome sequence data, we checked the original publications and only used data with key parameters equal or better than our own transcriptome assemblies (Additional file Table S1). Furthermore, we constructed multiple datasets with different levels of quality control. For our most strict dataset (dataset 1), we not only applied a strict automatic filtering process, but also manually checked all alignments one by one to confirm the quality. All our essential conclusions are derived from analysis of this dataset. For example, although convergent genes were initially identified by the pairwise analysis of datasets 1–7, only genes that were included in the dataset 1 were used for further analysis and discussion (e.g. Figure 2b). Furthermore, we conducted an additional round of manual quality check for all identified PSGs, FEGs, and convergent genes. Secondly, caution should be exercised when interpreting results from bioinformatic analysis. Such analysis may serve as an important first step; however, functional confirmation of the convergent mutations is essential to establish links to adaptation (see review [6]). The majority of the convergent amino acid substitutions are likely functionally inconsequential, such as in the case of convergent evolution of hemoglobin in high-elevation Andean waterfowls [8]. Thirdly, different detecting methods for molecular convergent evolution may yield quite different results. A better understanding of its null distribution is essential and better detecting methods are highly desirable.

## Conclusions

High-elevation adaptation may prove to be a fertile ground for studying molecular convergent evolution. Our examination detected several genes with large numbers of convergent and parallel sites, such as SULT6B1 and HSP90AA1. These genes are likely excellent targets for examining mutational bias, a primary cause of molecular convergent evolution. Other genes, such as IKZF5, which are under positive selection but have few convergent sites, are likely good candidates for functional tests. Furthermore, other levels of molecular convergent evolution, such as gene expression, allele frequency, functional units or regulatory pathways, and genetic architecture, should also be examined.

## Methods

### Species selection, data collection, and quality control

A total of 13 species were included in our analysis (Fig. 1), including four species from high elevations, eight of their low-elevation close relatives and one outgroup species. Data of three species were obtained from published genome data (*Rhinella marina*, NCBI assembly accession GCA 900303285.1; *Xenopus tropicalis*, NCBI assembly accession GCF 000004195.1; *Nanorana parkeri*, NCBI assembly accession GCF 000935625.1), data of six species were obtained from published transcriptome data (*Bufo gargarizans*, NCBI BioProject PRJNA383934; *Leptobrachium boringii*, NCBI Gene Expression Omnibus GSE89016; *Nanorana yunnanensis*, BIGD BioProject PRJCA000409; *Odorrana margaretae*, NCBI Sequence Reads Archive SRA091981; *Rana chensinensis* and *Rana kukunoris*, NCBI Sequence Reads Archive SRA060325). We sequenced transcriptomes for four species (*Bufo tibetanus, Oreolalax popei, Quasipaa spinosa,* and *Scutiger boulengeri*) in this study.

One male and one female of each species was collected between 2013 and 2015 (*B. tibetanus*: Mangkang, western China, N29.66963°, E98.37072°, collected on June 21, 2015; *O. popei*: Mt. Omei, western China, N29.35145°, E103.17482°, collected on June 3, 2013; *Q. spinosa*: Mt. Omei, western China, N29.56418°, E103.38918°, collected on July 1, 2013; *S. boulengeri*: Lake Yamdrok, Tibet, N29.11166°, E90.351074°, collected on June 25, 2014). All specimens were acquired and processed legally and ethically. Individuals were euthanized by immersion in MS-222 buffered solution (3 g/L), and tissue samples were collected and stored in Sample Protector Solution (TAKARA) immediately after euthanasia. For transcriptome sequencing, five tissues (brain, liver, heart, skeletal muscle, and gonad) were collected separately and RNA was extracted using Trizol protocol (Invitrogen, USA). We mixed the RNA from each tissue in approximately equal quantities for each species. This approach was aimed at maximizing the recovery of genes. The concentration and integrity of total RNA were examined using agarose gel electrophoresis, NanoPhotometer spectrophotometer (IMPLEN, USA), as well as Agilent Bioanalyzer 2100 system (Agilent Technologies, USA). The RNA integrity number (RIN) scores of the total RNA used for library preparation were greater than 8.3. The cDNA libraries were constructed and subsequently sequenced on an Illumina HiSeq2000 platform in Novogene Inc. (Beijing, China).

We then performed quality filtration and de novo assembly. The raw reads were first cleaned by filtering out the adapter sequences using Trimmomatic [69]. Reads with quality scores below Q20 in a five-base sliding window were trimmed. Low-quality bases (< Q15) at the leading or trailing end were also trimmed. Reads were discarded if any of the following three conditions were met: 1) the percentage of N or unknown base was larger than 10%; 2) the percentage of low-quality bases (< Q20) was higher than 50%; 3) shorter than 30 base pairs. Through this process, we obtained approximately 7G clean data of paired-end reads for each species. The assemblies were finally produced using Trinity [70] with default parameters according to the published protocols [71].

Putative orthologous genes were identified using the best reciprocal blast hits (BRBH) method with a low cutoff E value (10^− 10^). Well-annotated protein sets of *X. tropicalis* and *N. parkeri* were used as references to identify corresponding orthologs in other anurans. Only orthologs with best-hit scores across all pairs of species were kept. Amino acid alignments were generated using Clustal Omega [72] with its default parameters. Codon alignments were generated based on the amino acid alignments. To ensure the accuracy of the downstream analysis, 200 randomly sampled genes were selected and their alignments were manually checked. We used these alignments to establish appropriate criteria (e.g. sequence identity and alignment score) to filter unreliable alignments.

To serve different analyses, seven datasets were constructed. Dataset 1 includes orthologs shared by all 13 species. Dataset 2–7 each includes orthologs shared by two sets of species (one high-elevation species with two low-elevation relatives) and one outgroup species (*X. tropicalis*). Several analyses required particularly high-quality alignments, and an additional filtering process was applied to dataset 1. Strict criteria of > 60% sequence identity and > 2.0 alignment score based on BLOSUM62 was applied to all alignments. In addition, the flanking regions of all identified selective or convergent sites (ten amino acids on each side) were examined; if there was an insertion/deletion longer than 5 base pairs or if the similarity between any two sequences was lower than 40%, the alignment was excluded. Finally, all alignments in this dataset were manually checked and minor manual adjustments were applied to 19 alignments (1.73%).

### Phylogenetic analysis

Dataset 1 was used for this set of analyses. To reduce potentially detrimental effects of several confounding factors in phylogenomic reconstruction, such as non-phylogenetic signals [46] and natural selection [45], we used only fourfold degenerate sites (4D; all mutations produce synonymous changes) from the alignment of concatenated data. All gaps were removed using trimAl [73] with the ‘-nogaps’ option. The best-fit partitioning schemes and substitution models for the supermatrix were determined by the Bayesian Information Criterion in PartitionFinder [74]. Maximum likelihood trees were inferred using RAxML8 [75] and subsequently evaluated with 1000 bootstrap replicates.

### Detecting molecular convergent evolution

We first conducted pairwise comparisons using Zhang and Kumar’s [49] test. All datasets (1–7) were subjected to this test. Zhang and Kumar’s test calculates the probabilities that the observed convergent or parallel substitutions are attributable to random chance using a statistical method [49]. Both the Poisson model and the JTT-f_gene_ model were used. We did not use the JTT-f_site_ model as recommended by Zou and Zhang [29], because we had only seven species in these pairwise comparisons and the JTT-f_site_ model might produce a relatively large bias since the number of species analyzed was small [25]. Program Converg2 [49] was used for this analysis. We also examined convergent evolution between several pairs of low elevation species using the same method as background comparison [24]. These analyses are very sensitive and are prone to false positive [29], and therefore, they serve as preliminary testing.

We further calculated the ratios between the observed numbers and expected numbers of convergent and parallel sites (*R*_*conv*_ [29];) between high-elevation species and low-elevation species. This method requires high-quality data with a large number of species and we only applied this method to dataset 1. Expected total number of molecular convergent and parallel substitutions were estimated using the JTT-f_site_ model. The JTT-f_site_ model is much more conservative than the JTT-f_gene_ model used in the Zhang and Kumar’s test [29]. Furthermore, we compared these values of high-elevation species to those of the low-elevation species using the Wilcoxon test implemented in R [76]. A linear correlation between the expected convergent/parallel sites and observed convergent/parallel sites was estimated.

Finally, we conducted a convergent and divergent correlation test. The expected numbers of convergent and parallel sites are correlated with the number of divergent sites [19, 22]. The line of best fit of the correlation may serve as null expectation under neutral evolution. For this analysis, dataset 1 was used. Ancestral sequence reconstruction was carried out using the CODEML program from PAML [53]. Numbers of observed convergent sites and divergent sites were counted using our in-house script and plotted in R.

### Tests for positive selection among high-altitude anurans

We first tested for positively selected genes (PSGs) based on dN/dS ratio. This approach is considered conservative [77]. Accelerated evolutionary rate may also suggest positive selection [78, 79]. Therefore, we also tested for fast evolving genes (FEGs). Lastly, we tested for positively selected amino-acid sites. Dataset 1 was used for this set of analysis.

The branch-site model A [80] implemented in the program CODEML (in PAML, [53]) was used to detect positively selected genes (PSGs) along a specific lineage. Species tree based on 4D sites was used for CODEML and branch lengths were optimized on a gene-by-gene basis. All four high-elevation species were designated as “foreground” and tested simultaneously. We compared the alternative (dN/dS > 1) and the null (dN/dS = 1) models using a likelihood ratio test (LRT). A Chi-square test was conducted for each gene to assess statistical significance. The false discovery rate (FDR) method was applied to correct for multiple tests. For a gene, if the selection model has a significantly higher likelihood value than the neutral model does (FDR-adjusted *p*-value < 0.05), the gene is considered as having experienced positive selection along the foreground branch.

To identify FEGs, we first estimated evolutionary rates with 4D sites, 2nd codon position sites, and lineage-specific dN/dS ratios using dataset 1 (1098 orthologs). The absolute substitution rate of fourfold degenerate (4D) sites represents a neutral evolutionary rate, while absolute substitution rate of the second position in codons approximates an adaptive evolutionary rate. The absolute rate for each species was calculated with the r8s program [81]. Calibration times for each major node were collected from the TimeTree database (http://www.timetree.org/). Multiple calibration points provided overall more realistic divergence time estimates [82]. The penalized likelihood method and TN algorithm implemented in r8s was used to accommodate rate heterogeneity. The dN/dS is another widely used indicator of evolutionary rate for protein coding genes [83, 84]. The dN/dS ratio for each lineage was estimated using a maximum likelihood approach [85] implemented in CODEML (in PAML [53]). The free-ratio branch model, which allows the dN/dS ratio to vary for different branches, was applied to the concatenated supermatrix.

To identify the fast-evolving genes (FEGs) of high-altitude species, we ran the one-ratio branch model and multi-ratio branch model with CODEML to estimate the global and local dN/dS ratio, respectively [86, 87]. The one-ratio model assumes that all branches have been evolving at the same rate (null hypothesis), and the multi-ratio model allows foreground branch to evolve under a different rate (alternative hypothesis). All four high-altitude frogs were set as foreground. A likelihood ratio test was employed to compare the one-ratio branch model and the multi-ratio branch model [54]. The *p*-values of the chi-square test were adjusted by FDR correction for multiple tests. If a gene had an FDR-adjusted *p*-value < 0.05 and also a higher dN/dS in the foreground branch than in the background branch, it was considered a FEG in the foreground branch.

To identify individual amino acid sites under positive selection, we employed the mixed effects model of evolution (MEME) test [88] implemented in HYPHY [55]. Our purpose was to assess how many of the convergent and parallel substitutions were a product of positive selection, and therefore, only the 57 identified convergent genes from dataset 1 were subjected to this analysis.

### Functional prediction

Functional enrichment analyses were carried out using R package ‘clusterprofiler’ [89]. We primarily focused on Gene Ontology (GO) terms from “Biological Process (BP)” and “Molecular Function (MF)”. Significant over-representation was determined by FDR-adjusted p-value (< 0.05).

## Supplementary Information

**Additional file 1: Figure S1.** Relationships of upper elevation limit and evolutionary rate. (A) Rate derived from 4D sites. (B) Rate derived from second codon position sited (codon 2). (C) dN/dS ratio.

**Additional file 2: Table S1.** Summary information of the transcriptome assemblies used in this study.

**Additional file 3: Table S2.** Summarized results from Zhang and Kumar’s test of datasets 2–7.

**Additional file 4: Table S3.** Detailed results from Zhang and Kumar’s test of dataset 1. Six pairwise comparisons are conducted and all sites and genes experiencing convergent and parallel evolution are listed.

**Additional file 5: Table S4.** List of genes that were subjected to positive selection (PSGs).

**Additional file 6: Table S5.** List of genes experiencing fast evolution (FEGs).

**Additional file 7: Table S6.** List of amino acid sites that were subjected to positive selection.

**Additional file 8: Table S7.** Results from enrichment analysis of convergent and parallel genes based on dataset 1.

**Additional file 9: Table S8.** Results from enrichment analysis of convergent and parallel genes based on dataset 2–7.

**Additional file 10: Table S9.** Results from enrichment analysis of fast evolving genes (FEGs).

## Data Availability

All data have been deposited in NCBI (*B. tibetanus, Q. spinosa* and *S. boulengeri*, accession number PRJNA524747; *O. popei*, accession number PRJNA357944).

## Abbreviations

ATP6V1G1: ATPase H+ Transporting V1 Subunit G1
ATP6V1H: ATPase H+ Transporting V1 Subunit H
ANXA5: Annexin A5
BP: Biological Process
BRBH: Best reciprocal blast hits
dN/dS: Number of non-synonymous mutations/number of synonymous mutations
EEF1E1: Eukaryotic translation elongation factor 1 epsilon-1
FDR: False discovery rate
FEGs: Fast-evolving genes
GO: Gene Ontology
GLUL: Glutamate-Ammonia Ligase
GTR: General time reversible
Hb: Hemoglobin
HIF: Hypoxia-inducible factors
HSPA5: Heat Shock Protein Family A (Hsp70) Member 5
HSPA9: Heat Shock Protein Family A (Hsp70) Member 9
HSPD1: Heat Shock Protein Family D (Hsp60) Member 1
HSP90AA1: Heat Shock Protein 90 Alpha Family Class A Member 1
HS3ST1: Heparan Sulfate-Glucosamine 3-Sulfotransferase 1
JTT: Jones-Taylor-Thornton
ML: Maximum likelihood
MYBPC2: Myosin Binding Protein C, Fast Type
IKZF5: IKAROS Family Zinc Finger 5
LRT: Likelihood ratio test
MF: Molecular Function
PSGs: Positively selected genes
RAP2A: RAP2A, Member of RAS Oncogene Family
RDH16: Retinol Dehydrogenase 16
RH1: Rhodopsin RPS12
SOD3: Superoxide Dismutase 3
STARD10: START domain-containing protein 10
SULT6B1: Sulfotransferase Family 6B Member 1
TACC3: Transforming Acidic Coiled-Coil Containing Protein 3
TMEM238: Transmembrane Protein 238
TTX: Tetrodotoxin
UV: Ultraviolet
