# Supplementary material for: Molecular convergent and parallel evolution among four high-elevation anuran species from the Tibetan region
Source: BMC Genomics. 2020 Nov 27;21:839. doi: 10.1186/s12864-020-07269-4 (PMC7694343; doi:10.1186/s12864-020-07269-4)
Supplement: Supplementary file 1 — Additional file 1: Figure S1. Relationships of upper elevation limit and evolutionary rate. (A) Rate derived from 4D sites. (B) Rate derived from second codon position sited (codon 2). (C) dN/dS ratio. [file 12864_2020_7269_MOESM1_ESM.pdf]

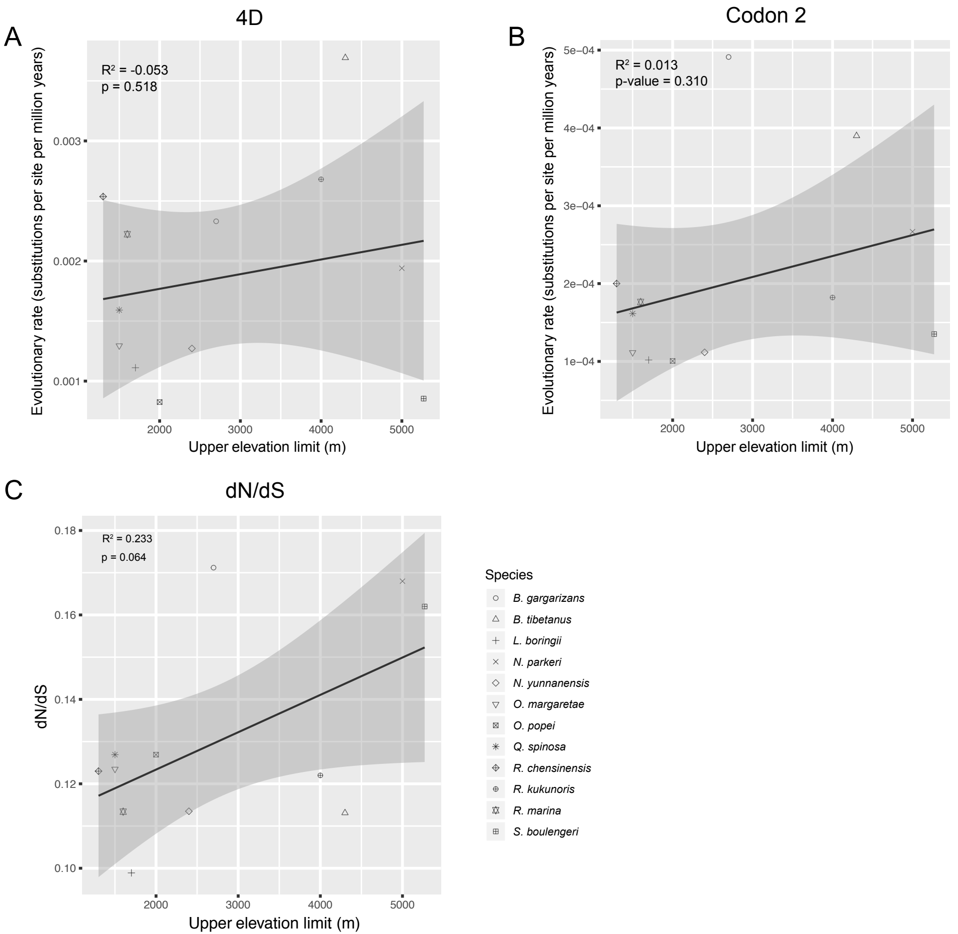
A
4D
R² = -0.053
p = 0.518
Evolutionary rate (substitutions per site per million years)
0.003
0.002
0.001
2000
3000
4000
5000
Upper elevation limit (m)
B
Codon 2
R² = 0.013
p-value = 0.310
5e−04
4e−04
3e−04
2e−04
1e−04
Evolutionary rate (substitutions per site per million years)
Upper elevation limit (m)
C
dN/dS
R² = 0.233
p = 0.064
0.18
0.16
0.14
0.12
0.10
dN/dS
Upper elevation limit (m)
Species
B. gargarizans
B. tibetanus
L. boringii
N. parkeri
N. yunnanensis
O. margaretae
O. popei
Q. spinosa
R. chensinensis
R. kukunoris
R. marina
S. boulengeri
